# Optimizing Angioplasty in Elderly Patients with Multimorbidity: A Paradigm Shift in Risk Assessment: Optimizing Angioplasty in Elderly Patients with Multimorbidity

**DOI:** 10.31661/gmj.v14i.3768

**Published:** 2025-03-01

**Authors:** Amir Ahmadian Hosseini, Ahmad Separham

**Affiliations:** ^1^ Cardiovascular Research Center, Tabriz University of Medical Sciences, Tabriz, Iran

**Keywords:** Angioplasty, Elderly Patients, Frailty, Percutaneous Coronary Intervention

## Dear Editor,

The growing number of elderly patients undergoing angioplasty, coupled with high rates of multimorbidity and frailty, presents significant clinical challenges [1]. While percutaneous coronary intervention (PCI) remains a cornerstone for managing coronary artery disease (CAD), conventional risk scores such as GRACE often fail to incorporate age-specific factors like frailty, functional decline, and polypharmacy, which critically influence outcomes [2][3] a potential advantage of adding the elements of geriatric assessment to the commonly used Global Registry of Acute Coronary Events (GRACE). Evidence increasingly highlights the need for more nuanced risk assessment strategies to improve procedural decision-making and patient outcomes [4]. This paradigm shift moves beyond traditional, static risk scores to a dynamic, multi-dimensional approach that reflects the unique vulnerabilities and goals of elderly patients [3]. In patients with multimorbidity such as diabetes, chronic kidney disease, and heart failure balancing procedural benefits and risks becomes particularly complex [5]. While angioplasty improves symptoms and functional capacity, elderly patients face heightened risks of contrast-induced nephropathy (CIN), bleeding, and prolonged hospital stays, especially when multiple comorbidities coexist [4]. However, a recent study by Mele et al. [6], demonstrated frail patients treated with PCI had significantly lower mortality rates at both 30 days (17%) and 6 months (37%), compared to same subjects who did not undergo PCI (48% and 71%, respectively) [6]. Similarly, a systematic review and meta-analysis by Wang et al. [5] demonstrated that integrating frailty indices improved risk prediction for bleeding complications and mortality.

Despite the presence of greater comorbidities in elderly patients, advancements in angioplasty techniques, such as the shift to radial artery access, as the standard approach for routine coronary procedures in older patients seem practical and safe [7]. Moreover, next-generation drug-eluting stents (DES), particularly ultrathin-strut DES, have demonstrated a 15% reduction in the risk of target lesion failure during long-term follow-up [8]. Coupled with precision-driven technologies like intravascular ultrasound (IVUS) and optical coherence tomography (OCT), these advancements ensure safer and more effective PCI, particularly in elderly patients with multimorbidity [9].

Practical barriers to implementing frailty assessments, such as time constraints and lack of standardized tools, remain a challenge [5]. However, streamlined approaches using short frailty screening tools, such as gait speed tests or the "Timed Up and Go" test, offer feasible solutions. These tools are quick take under 5 minutes and require minimal training, making them suitable for busy clinical settings [3][10] 2017 and December 31st, 2020. Frailty was retrospectively assessed using the Canadian Study and Aging Clinical Frailty Scale (CFS. Also, integrating digital tools and automated frailty scoring systems into electronic health records can streamline assessments, improving workflow efficiency and standardization across institutions [11].

To facilitate this paradigm shift, clinical decision-making must integrate frailty assessments, multimorbidity risk profiles, and technological advancements into a cohesive framework [6]. A patient-centered approach, emphasizing shared decision-making, remains critical to align treatment strategies with individual goals and values, particularly for patients with limited life expectancy or severe functional decline [10] 2017 and December 31st, 2020. Frailty was retrospectively assessed using the Canadian Study and Aging Clinical Frailty Scale (CFS).

In conclusion, by combining frailty evaluations, addressing multimorbidity through targeted strategies, and leveraging modern PCI techniques, clinicians can deliver safer, more personalized care for elderly patients. By embracing this integrated framework, clinicians can transform PCI outcomes for elderly patients, ensuring safer, more effective, and patient-centered care that meaningfully improves both quality of life and procedural success.

## Conflict of Interest

The authors have no competing interests to declare that are relevant to the content of this article.
